# Reading Deficits in Intellectual Disability Are still an Open Question: A Narrative Review

**DOI:** 10.3390/brainsci8080146

**Published:** 2018-08-07

**Authors:** Francesco Domenico Di Blasi, Serafino Buono, Santina Città, Angela Antonia Costanzo, Pierluigi Zoccolotti

**Affiliations:** 1 Oasi Research Institute—IRCCS, Via Conte Ruggero, 73, 94018 Troina, Italy; fbuono@oasi.en.it (S.B.); scitta@oasi.en.it (S.C.); acostanzo@oasi.en.it (A.A.C.); 2 Department of Psychology, Sapienza University of Rome, Via dei Marsi 78, 00185 Rome, Italy; pierluigi.zoccolotti@uniroma1.it; 3 ISTC Institute for Cognitive Sciences and Technologies, CNR, 00185 Rome, Italy

**Keywords:** intellectual disability, reading, genetic syndrome

## Abstract

Background. In children with intellectual disability (ID), the acquisition of reading skills constitutes a basic step towards the possibility of independent living, social inclusion and participation. Methods. We carried out a narrative review of the literature on reading fluency and accuracy of individuals with ID resulting from different genetic syndromes (Fragile X, Williams, Velocardiofacial, Prader-Willi, and Down syndrome). Our aim was to define their reading profiles in light of the dual-route reading model. For this purpose, studies that examined both word and non-word reading in children with ID were included in the analysis. Results. Seventeen studies emerged based on the selection criteria. The results were different depending on the control group used. A deficit in reading non-words emerged in studies that used the reading-level match design but not when standardized scores were used, when controls were age-matched or when a mental age matching was used. Thus, a deficit in reading non-words emerged only in studies that used the reading-level match design. However, severe methodological criticisms were recently raised about the use of this matching design. Conclusions. In view of the methodological problems in using grade equivalents, it is premature to draw definite conclusions about the reading profile of children with ID resulting from different genetic syndromes. In any case, the reviewed evidence provides little support for the idea that children with ID have selective difficulty in phonological reading. Thus, the reading profile of children with ID remains an open question that needs to be investigated by means of methodologically sound research.

## 1. Introduction

For children with intellectual disability (ID), the acquisition of reading skills is a basic step in creating solid foundations for school achievements, reaching a sufficient level of independence and creating an adequate quality of life. Therefore, the lack or reduced acquisition of these skills, as well as their defective automatization, impedes school learning and may become a barrier for social participation [1,2].

Learning to read is accomplished through the acquisition of procedures that have limited degrees of freedom and, consequently, can be automatized and measured. For example, word identification (or decoding) occurs through the learning of correspondences between signs and sounds in a unitary fashion. The possibility of pronouncing a given word in different ways is defined by grapheme-to-phoneme conversion rules (although adherence to rules partially varies according to the characteristics of the orthography). Subsequently, the constant experience of reading allows for a quick association between sound combinations and orthographic word characteristics and thus fosters the acquisition of an orthographic input lexicon. 

However, learning to read does not become automatic for everyone and individuals with ID with different levels of development may reach different levels of reading skills. Some students with significant cognitive disabilities in “functional” reading (global word reading) may reach a basic level of alphabetization [3]. Others may master the decoding components (i.e., lexical-orthographic and/or phonological) better and even reach higher levels than expected for their mental age or IQ level; however, this is not true for the reading comprehension component [4]. 

In our review, we aimed to analyze the reading skills of children/adolescents with ID due to different genetic syndromes, both in terms of accuracy and fluency (defined as the ability to read quickly) [5,6]. One important theoretical framework for interpreting reading proficiency is provided by the dual-route reading model [7]. According to this model, reading can be accomplished through a direct-lexical route (in which words are recognized as wholes with reference to entries in the orthographic lexicon) or on the basis of an indirect sub-lexical, phonological route (which carries out the conversion between graphemes and phonemes). Previous studies on children/adolescents with ID emphasized the presence of a phonological deficit, as indicated by the presence of a deficit in reading non-words (for reviews see [8,9]), as well as a deficit in phonological awareness (for a review see [10]). Based on this evidence, up-to-date reading interventions in children/adolescents with ID has primarily focused on a phonics approach aimed at fostering phonological skills (for reviews, see [11,12]). However, there is some reason to question the general conclusion that ID is specifically associated with fragility of the sub-lexical phonological route. Recent research indicates that the reading-level match approach frequently used to evaluate reading efficiency is inherently flawed [13,14]. In particular, it allows for the emergence of a deficit for stimuli/conditions for which one would expect a slower developmental path, for example, the case of non-word reading. These methodological concerns are spelled out in more detail in Section 3.1. In any case, the presence of a potential bias in studies based on the reading-level approach underscores the importance of separately evaluating studies using this paradigm.

The focus of our review is to examine the possible moderating role of different genetic syndromes. Among the genetic conditions we chose to focus our search on, some of the most frequent ones are Fragile X syndrome, Williams syndrome, Velocardiofacial syndrome, Prader-Willi syndrome and Down syndrome. Below, we briefly describe the main characteristics of these syndromes including their known cognitive correlates. 

Fragile X syndrome (FXS) is caused by the transcriptional silencing of the *FMR1* gene (Xq27.3) due to the progressive expansion and subsequent methylation of (CGG) trinucleotide repeats in the 5′-untranslated region of the gene [15]. In males, the full mutation of the *FMR1* gene can be associated with mild-to-severe intellectual disability; in females, the level of ID is usually mild. FXS presents with a variable clinical phenotype: psychomotor and language developmental delay, problems with working and short-term memory, executive function, language, mathematics and visuospatial abilities, anxiety, mood instability, autism, hyper-arousal, attention deficit hyperactivity disorder; recurrent otitis and seizures can also be observed [16,17,18]. 

Williams syndrome (WS) is a rare genetic multi-systemic neuro-developmental disorder caused by deletion on the long arm of one of two chromosomes in chromosome pair 7 (7q11.23) [19]. WS is characterized by a distinctive facial appearance, cardiac anomalies, stature deficiency, characteristic dental malformation, infantile hypercalcemia, fine and gross motor developmental delay, cognitive abnormalities, atypical sleep pattern and connective tissue abnormalities. Most individuals with WS have an uneven cognitive profile. For example, many non-linguistic functions, such as spatial cognition, planning and problem-solving and visuo-motor integration, which cause problems in drawing, writing and mathematics, are significantly impaired [20,21]. On the other hand, good phonology and performance in the normal range on short-term verbal memory tasks are also found. However, these individuals also have deficits in some specific aspects of oral language, such as vocabulary, speech and language comprehension, morpho-syntax and semantics [22]. Characteristic behavioral traits include the tendency to be extremely talkative and over-friendly with strangers, overstepping conventional social boundaries, anxiety, difficulties with social interaction and verbal and non-verbal communication, autism spectrum disorders and attention deficits [23].

Velocardiofacial syndrome (VCFS) is in most cases due to a deletion on chromosomal region 22q11.2. The deletion is due to a non-allelic meiotic recombination during spermatogenesis or oogenesis [24,25]. VCFS causes a congenital malformation disorder and shows a variable clinical phenotype that can range from mild to severe. Common features include cardiac defects, palatal anomalies, facial dysmorphism, vertebral anomalies, developmental delay and immune deficiency. Additional clinical findings may include gastrointestinal anomalies, hearing loss, renal anomalies, dental anomalies, mild to moderate intellectual disability, attention deficit hyperactivity disorder, autism, anxiety and schizophrenia [26,27].

In approximately 70% of cases, Prader-Willi syndrome (PWS) is caused by a deletion on the paternally derived chromosome 15; in 25% of cases, PWS is due to maternal uniparental disomy (UPD) and 5% of cases are caused by to an imprinting defects and atypical deletions [28]. The disease is clinically heterogeneous. Common features include delayed psychomotor and language development, severe hypotonia, various degrees of intellectual disability, hypothalamic-pituitary abnormalities, dysmorphic facial appearance, compulsivity, behavioral problems, hyperphagia, obesity, autism and psychosis. However, some of these characteristics may vary in the distinct genetic subtypes [29,30,31].

Down syndrome (DS) is the most common neurodevelopmental disorder of known genetic origin. The chromosomal abnormality is caused by the presence of a third (partial or total) copy of chromosome 21 [32]. This chromosomopathy generates muscular hypotonia, joint laxity and specific morphological features. Malformations and complications include: short stature, congenital cataract, conductive hearing loss, heart defects, digestive malformations, Hirschsprung disease, epilepsy, sleep apnea, sensory deficiencies, leukemia, auto-immune and endocrine pathologies, diabetes mellitus type 1, alopecia areata, earlier aging, Alzheimer’s disease, and variable and often mild-to-moderate intellectual disability. The cognitive profile varies widely with relative strengths in visuospatial processing and implicit long-term memory and more difficulty in short-term verbal memory, long-term visual memory, expressive language and executive function [33,34,35,36]. 

Even from this brief overview, it is clear that there is substantial variability in the cognitive profile associated with these syndromes which may also be reflected in variable reading performance. In particular, it is of note that some genetic conditions are frequently associated with language delay and, more in particular, with problems in phonological processing and short-term memory (the FXS, PWS and DS syndromes), whereas others are characterized by a relative sparing of these skills (in particular the WS syndrome). The presence of phonological deficits in some, but not all, genetic syndromes examined may be relevant in view of the hypothesis of a fragility of the sub-lexical phonological route in the reading of children with ID [4,8,9]. In our review, we aimed to examine the reading skills of individuals with ID due to different genetic syndromes, and to define a reading profile in relationship to the dual-route reading model; for this purpose, only studies that examined both word and non-word reading were included in the analysis [7].

## 2. Method

### 2.1. Study Design

Review of the literature published before October 2017.

### 2.2. Search Strategy

The following databases were used: Pubmed/MEDLINE, PsycINFO, Cochrane Library, Ebscohost, Proquest. The search was carried out using the following term combination: (“reading” OR “decoding” OR “literacy” OR “fluency” OR “pseudoword” OR “nonword” OR “irregular word”) AND (“Fragile X” OR “Down syndrome” OR “Williams syndrome” OR “Velocardiofacial” OR “Prader-Willi syndrome”).

### 2.3. Selection Criteria

We included studies that (a) reported original research, (b) were conducted in groups of patients with genetic syndromes, (c) were published in English in peer-reviewed journals, and (d) used standardized tests of word decoding (accuracy and/or speed) and of non-words (accuracy and/or speed) compared to normative data or to the performance of a control group.

Papers not consistent with the area of interest or with the selection criteria, such as review papers, comments, editorials or letters, were not taken into consideration.

### 2.4. Selection, Procedures, Screening and Data Elaboration

**Figure 1:**
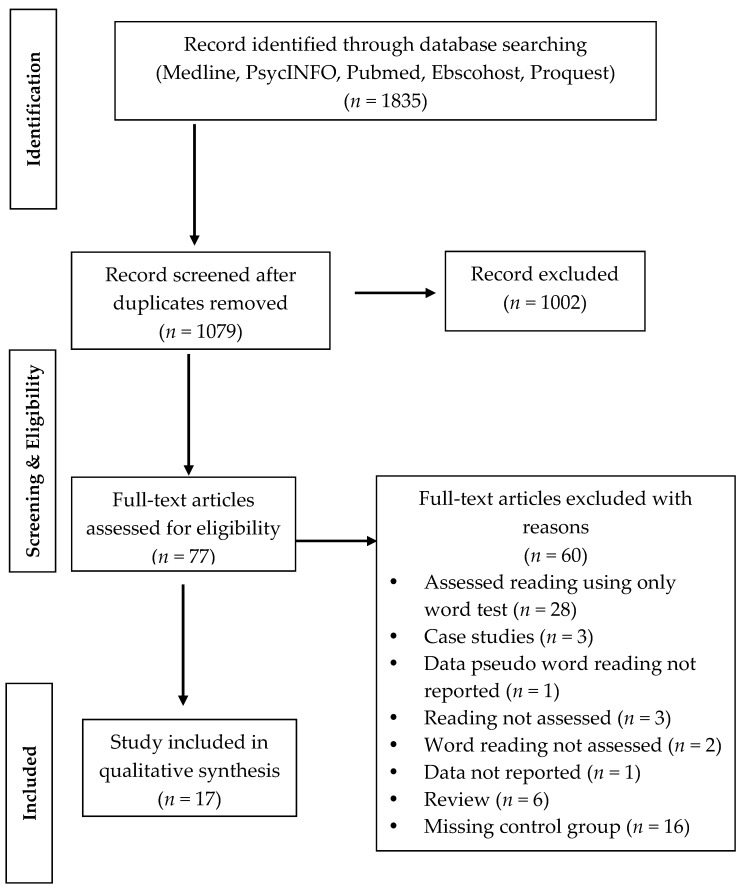
Flow diagram of selection process of studies.

The process of searching and selecting papers was carried out by two of the authors (F.D.D. and S.B.) and that of determining the eligibility of papers was carried out by two other independent authors (A.M.C. and S.C.). The research algorithm produced a total of 1835 studies, 77 of which were considered to be potentially eligible (see Figure 1). For Prader-Willi syndrome, we did not find any peer-reviewed studies that examined reading skills in an in-depth fashion; thus, no study on this condition was analyzed.

## 3. Data Synthesis

Out of the 77 potentially eligible studies, we found a total of 17 studies; 60 were not included as they did not meet the selection criteria (see Figure 1). Considering the limited number of studies included, we aimed to make a narrative synthesis, which included the description, organization, exploitation and interpretation of the studies, considering their methodological adequacy. Details about the methodology and results of the single studies are presented in Table 1. 

### 3.1. Methodological Considerations in Control Group Selection

As stated in the Introduction, recent research has indicated a potential methodological pitfall in studies based on the mental-age or reading-level match [13,14]. Reference to age equivalents has been widely used in cognitive research in general, and in reading research in particular [54,55,56]. The aim of these designs is to compare individuals with different pathological conditions to groups of individuals with relatively similar performance to detect areas of strengths and weaknesses. However, severe methodological criticisms have been raised about the use of these paradigms [13,57]. The critical point here is that using a reading-level matched control group is based on the assumption that the acquisition of the skills being compared is homogeneous across development. However, this assumption is quite unlikely, in view of the fact that only the words are by definition practiced by children. Indeed, there is evidence that word and non-word reading have different developmental trajectories [58]. In itself, this violates the assumption that the cognitive skills being compared have a homogeneous developmental acquisition, introducing a severe bias in the analysis. In particular, a deficit may emerge for skills that develop more slowly than the control task and/or for tasks that are generally more difficult (and/or produce greater levels of inter-individual variability) [14]. In this vein, the use of mental-age or reading-level matched control groups is likely to introduce severe biases in the data. 

As stated above, there is also a long tradition of studies using this approach in reading research. Three different reviews have summarized the relevant studies [59,60,61]. The general finding is that children with dyslexia show a deficit in non-word reading, in comparison to control children matched for reading level, usually (though not always) in terms of their word decoding capacity. This deficit has been instrumental in interpreting dyslexic symptoms. In general, in all of these reviews, it was concluded that, as children with dyslexia are selectively impaired in reading non-lexical stimuli, dyslexia should be specifically associated with a deficit in phonological skills [59,60,61]. However, van den Broeck and Geudens [14] have shown that this putative deficit is an artifact of the matching procedure. As non-word reading is generally more difficult (and associated with greater inter-individual variability), a comparison based on reading level matching is biased toward finding a putatively selective deficit in non-word reading. If paradigms specifically aimed to control for such a bias are used (such as the state trace analysis in the case of the study by van den Broeck and Geudens [14]), there is no evidence that children with dyslexia are specifically impaired in non-word reading. Procedures such as the state trace analysis, originally developed by Bamber [62], are infrequently used in clinical studies. However, reference to standardized zeta scores (based on norms from chronological peers) is a more standard procedure that is not sensitive to the bias introduced by the use of mental- or reading-level matching [14]. 

Based on the above considerations, in analyzing the results of the literature we will present separately the results obtained by studies using (a) the mental-age match design, (b) the reading-level match design and (c) those using standard scores or chronological age match. We believe that information on the type of control group used is important in interpreting the results obtained.

## 4. Results

### 4.1. Studies Based on the Mental-Age Matching Design

Two studies on children with FXS [37,38] and two on children with WS [39,41] used mental age matching in their design and found that these children performed better than controls in reading words, but they were not different with respect to non-words. In one study [40], the performance of children with WS was found to be generally impaired: reading of words was comparable to that of the control group and reading of non-words was lower (although only in accuracy, not speed). Unique in the present review, the study by Johnson-Glenberg [37] included a control group matched for nonverbal mental age and one for reading level (children in the latter group were of an intermediate age between that of FXS individuals and of mental age matched controls). When the comparison was made based on mental age, the children with FXS performed better in word reading (in line with most previously quoted studies). When the comparison was made in terms of reading level, they were impaired in non-word reading with respect to controls (note that word reading performance was comparable as it was used in the matching procedure).

Therefore, the results of studies that relied on mental-age matched controls generally indicated that (a) reading is relatively spared compared to general cognitive abilities (intelligence) and (b) non-word reading is more affected than word reading and, in most studies, is not distinguishable from that of mental age-matched controls.

### 4.2. Studies Based on the Reading-Level Design

We found several (*n* = 10) studies that used the reading level matching procedure but they all referred to children with DS. All studies were informative in terms of non-word reading but only a few were for word reading as this latter test was often (though not always) used to match the groups with and without DS. 

With this caveat, ten of the studies in Table 1 present results on the non-word reading performance of children with DS. Six of these studies [47,48,49,50,51,53] found an impairment in non-word reading compared to reading level-matched controls but four [44,45,46,52] did not. Only a few studies selected groups based on measures other than word reading. For example, Gombert [45] matched children on a measure of text reading and then tested performance on word reading and found no group difference.

Therefore, results from the reading-matched studies are mixed, but in most cases a selective deficit for the reading of non-words was detected in children with DS.

### 4.3. Studies Based on Standard Zeta Scores or Chronological Match

We found only two studies that did not rely on reading- or mental-age comparisons. Both studies examined the reading skills of children and young adults with VCFS. 

The study by Swillen et al. [42] explored the level of learning, the psychosocial characteristics and the neuropsychological profile of a small group of children with a mean FSIQ of 74 (SD = 3.7) and a mean age of 10 years and five months (age range 6.10–12.10). The reading capacities found in the decoding of words (mean *z*-score = 0.043, SD = 0.911) and non-words (mean *z*-score = −0.05, SD = 0.881) were very close to the mean of the normative sample and did not reveal a difference between lexical and non-lexical stimuli. 

The study by Lewandowski et al. [43] focused on how associated psychiatric conditions contribute to defining the neurocognitive profile of children and adolescents with VCFS, and also included an evaluation of reading, spelling and mathematics. The children had a mean FSIQ of 70.7 (SD = 12.4) and a mean age of nine years and three months (age range 7–16 years). They were compared to a control group with the same age and gender composition. Compared to controls, the children with VCFS were impaired in a global measure of reading, in reading words, in word comprehension and spelling. However, they were not impaired in non-word reading. Clear-cut differences were observed in the mathematical tasks (with larger effect sizes than reading and spelling). This finding is consistent with some suggestions in the literature [63] that the performance of these children is better in reading than in numerical tasks. 

## 5. Discussion

In the present review, we set out to examine the reading skills of individuals with ID due to different genetic syndromes. The acquisition of reading skills in these children is a basic step for school achievement and understanding the nature of their difficulty can be instrumental to more effective and individually tailored interventions when needed.

Based on recent methodological work [13,57], we expected that the results might be sensitive to the type of design used (i.e., based on the mental-age match design, the reading-level match design or on a comparison with standard scores or chronological age match). The results confirmed this expectation in that different results were obtained based on these different control groups. Thus, studies based on mental age matching generally found that children with ID performed better in reading words than controls but did not perform differently when reading non-words. Most (though not all) studies that used the reading level match found a selective deficit for the reading of non-words in children with ID (all studies were actually based on the examination of children with DS). By contrast, the only two studies that used either standardized scores or a chronological match failed to find any deficit in reading non-words while results for word reading were variable. One study [42] found no deficit in children with VCFS while the other [43] found small group differences. Further support for the idea that the type of control group is critical in producing different outcomes derives from the study by Johnson-Glenberg [37], which included both a control group matched for nonverbal mental age and one for reading level: in the former case, children with FXS scored higher in word reading than controls while, in the latter, a deficit in reading non-words emerged.

One of the aims of this review was to evaluate the possible differences in reading skills of children with different genetic syndromes. We found that the use of different experimental designs was actually nested with the type of genetic condition. Thus, most studies with the reading level match were carried out in children with DS, whereas those based on standardized scores or chronological matching examined children and young adults with VCFS. As different methods yielded different results, it is difficult to tease out the role of the specific genetic syndrome, if any, over and above the influence of the paradigm used.

In general, the reviewed evidence provides little support for the idea that children with ID have selective difficulty in phonological reading. Indeed, a deficit in reading non-words emerged only in studies that used the reading-level match design; it was not apparent when standardized scores were used [42], when controls were age-matched [43] or when a mental age matching was used [37,38,39,40,41]. In the reading age level design, children with ID are matched to controls on word reading and the focus is on group differences in non-word reading. However, it has been shown that this design makes the unlikely assumption of homogeneity of development [13]. By contrast, it is well known that word and non-word reading follow different developmental trajectories; thus, performance in reading non-words develops more slowly than reading words and it generates greater inter-individual variability than word reading [58]. It has been demonstrated that either of these characteristics is sufficient to generate an apparent deficit in non-word reading when using the reading level match [14].

One general question concerns whether reading is an area of relative strength or weakness in children with ID. Although this was not the specific aim of the present analysis, the reviewed evidence seems to indicate that reading may not be the most affected area in these children. In fact, various studies indicated that children with ID generally performed better in word reading than children matched for mental age. Furthermore, studies using standardized scores or a chronological match design either found no group differences [42] or group differences [43] that were actually smaller than those found for mathematical tasks [43]. Overall, evidence indicates that, although difficulties are certainly present in children with ID, reading may actually be a relatively spared area in at least some of these children.

Overall, it appears that the available evidence is insufficient to allow for the drawing of definite conclusions about the reading profile (in terms of lexical vs. non-lexical deficits) of children with ID and, in particular, that it is too early to identify possible differences in the reading profile of children with different single genetic syndromes. Albeit “negative”, we feel that this statement is indeed important because we hope it will raise awareness about the importance of new studies that are immune from methodological problems so as to have more valid and coherent information on the phenomenon.

However, the present observations raise the possibility that the reading profile of children with ID might be quite different from what has been believed so far, and in particular, that there is no strong reason to endorse the presence of a sub-lexical reading deficit in children with ID [4,8,9]. As reading (and writing) are key aspects of human functioning and are essential if schooling is to be effective, there seems to be a clear need for studies that examine reading performance in children with ID resulting from different pathological conditions. For this purpose, it should be noted that standardized zeta scores are not sensitive to the bias introduced by the use of mental- or reading-level matching designs [50,51]. Thus, we propose that the use of this approach in future research might allow for the obtaining of reliable information about the reading skills of children with ID.

## 6. Implications for Clinical Practice and Research 

It is crucial to test word and non-word reading to determine whether a child has problems in the lexical or in the phonological reading route. Until now, the over-representation in the literature of studies carried out with the reading-level design has brought to light that children with ID (and particularly with DS) are impaired in phonological processing [4,8,9]. Consistently with these findings, it was suggested that a phonics approach [11,12] be adopted in the rehabilitation programs developed for these children. Based on the results of the present review, it seems that further research is needed before a definite conclusion can be reached on this point.

## 7. Limitations

The limitations of the present analysis are largely due to the presence of a biased database with a predominance of studies using the mental-age or reading-level matching design to recruit control groups. In view of the pitfalls that have been convincingly demonstrated in these paradigms [13,14,57], the available evidence is insufficient to draw firm conclusions about the reading skills of children with ID. Although this is certainly a limitation, considering the importance of this behavior for the overall development and adjustment of these children, we hope that the paper will generate new interest in carrying out research on these children that is not methodologically biased.

## 8. Conclusions

Our review of studies on the reading skills of children with ID due to genetic syndromes does not allow us to reach firm conclusions. In particular, the previously reported [4,8,9] greater involvement of non-lexical over lexical reading appears artefactual and due to a statistical bias introduced by the use of control groups selected on the basis of a reading-level matching. Thus, there is a need for studies that are free from this methodological pitfall and provide more valid and coherent information on the phenomenon. This consideration has important clinical implications; in particular, it indicates that emphasis on phonological (or meta-phonological) exercises in the training of reading of children with ID is not warranted based on the available evidence. Therefore, the nature, and to some extent, the presence of reading deficits in children with ID are still open questions. Indeed, case reports of children with severe ID and brilliant reading skills have been reported [64,65], but it seems that the lesson from these studies has been over-looked.

## Figures and Tables

**Table 1:** List of selected studies. FXS = Fragile X Syndrome. WS = Williams Syndrome. VCFS = Velocardiofacial Syndrome. DS = Down Syndrome.

	Reference	*n*	Age at Assessment M (SD) Years	IQ	Decoding Skill Measures	Group-Matching Design	Decoding Skills: Findings
FXS	Johnson-Glenberg (2008) [37]	*n* = 15 males with FXS *n* = 22 TD males *n* = 13 TD males	Mean age = 20.6 (range 11.5–38.1) Mean age = 5.5 (range 3.11–8.3) Mean age = 7.6 (range 5.4–8.3)	Mean mental age = 5.30 (SD = 1.26) (Stanford-Binet)	Accuracy: word identification (WRMT-R) word attack (WRMT-R)	Mental-age Word reading-age	FXS > TD mental age group on word identification TD age word reading group > FXS on word attack
FXS	Klusek et al. (2015) [38]	*n* = 51 males with FXS *n* = 35 TD males	Mean age = 10 (range 7.9–13.2) Mean age = 5.1 (range 3.3–7.4)	IQ = 56 (36–74) mean mental age = 5.4 (SD = 0.6) (range 4.1–6.7) (Leiter-r)	Accuracy: letter-word identification (WJ-R) word attack (WJ-R)	Mental-age	FXS > TD on letter-word identification FXS-TD = NS on word attack
WS	Laing et al. (2001) [39]	*n* = 15 males with WS *n* = 15 TD males	Mean age = 15.1 (range 9–27.7) Mean age = 6.9 (range 5–9.2)	General Cognitive Ability = 43.8 (SD = 5.12) (range 39 to 54) (BAS)	Accuracy: single word reading (WORD); nonword reading (GNRT)	Mental-age	WS > TD on word reading WS-TD = NS on nonword reading
WS	Menghini et al. (2004) [40]	*n* = 16; 10 males, 6 females with WS *n* = 16; 10 TD males, 6 TD females	Mean age = 17.7 (range 10.9–30.2) Chronological age range 6.2–8.6	Mental age = 7 (range 5.2–10.2) (Stanford-Binet)	Accuracy: word reading nonword reading Speed: word reading nonword reading (DDE)	Mental-age	TD > WS on accuracy nonword; WS-TD = NS on word accuracy and on word and nonword speed
WS	Garayzabal-Heize et al. (2008) [41]	*n* = 12; 5 males, 7 females with WS *n* = 12; 6 TD males, 6 TD females	Mean age = 12.4 (range 8–15) Mean age = 7.9 (range 6–9)	Mean IQ = 50.6 (WISC) Mean agemental verbal = 7.8 (PPVT)	Accuracy: word reading nonword reading Speed: word reading nonword reading (PROLEC-R)	Mental-age	TD > WS on accuracy and speed word; TD-WS = NS on accuracy and speed nonword;
VCFS	Swillen et al. (1999) [42]	*n* = 9; 4 males, 5 females with VCFS	Mean age = 10.5 (range 6.10–12.10 years)	Mean FSIQ = 74 (SD = 3.70) (WISC-R)	Accuracy and speed: word decoding (Brus-EMT) Accuracy and speed: Klepel-Pseudoword-test	Raw scores obtained on each of the tests were standardized using published age-based norms (*z*-scores)	Mean *z*-scores word and pseudoword decoding = age-based norms.
VCFS	Lewandowski et al. (2007) [43]	*n* = 26, 16 males, 10 females with VCFS *n* = 25, 13 males, 12 females TD	Mean age = 9.3 (SD = 2.6) (range 7–16 years) Mean age = 9.7 (SD = 2.4) (range 7–16 years)	Mean FSIQ = 70.7 (SD = 12.4) (WISC-III)	Accuracy and automaticity: word reading (WIAT-II) Accuracy: pseudoword decoding (WIAT-II)	Chronological age and gender	TD > VCFS on word reading VCFS-TD = NS on pseudoword decoding
DS	Cossu et al. (1993) [44]	*n* = 10 DS *n* = 10 TD	Mean age = 11.4 (range 8–15.8 years) Mean age = 7.3 (range 6.9–7.9 years)	IQ = 44 (range 40–56) (WISC)	Accuracy: word reading nonword reading	Reading-level	TD-DS = NS on nonword reading
DS	Gombert (2002) [45]	*n* = 11 DS *n* = 11 TD	Mean age = 13.9 (range 10.5–20.0 years) Mean age = 7.1 (range 6.6–8.1 years)	IQ = 47 (range 44–50) (WISC)	Accuracy: word reading irregular word reading; nonword reading; (neighbor and non-neighbor)	Reading-level	TD-DS = NS on accuracy word and irregular reading TD-DS = NS on neighbor and non-neighbor nonword reading
DS	Snowling et al. (2002) [46]	*n* = 29 DS *n* = 31 TD	Mean age = 13.2 (range 6.11–17.6 years) Mean age = 5.3 (range 4.6–6.5 years)	Vocabulary: age equivalent in months = 4.1 (range 1.09–8.07) (BPVS)	Accuracy: single word reading (BAS) nonword reading (GNRT)	Reading-level	TD-DS = NSon nonword reading
DS	Verucci et al. (2006) [47]	*n* = 17 DS *n* = 17 TD	Mean age = 16.5 (range 7.7–28.5 years) Mean age = 7 (range 6.2–8.6 years)	Mental age = 6.2 (range 5.2–7.8)	Accuracy: word reading nonword reading Speed: word reading nonword reading (DDE)	Reading-level	TD > DS: on accuracy nonword reading TD-DS = NS on speed nonword reading
DS	Kay-Raining Bird et al. (2008) [48]	*n* = 20 DS *n* = 17 TD children	Mean age = 171.7 months (range 8.6–19.10 years) Mean age = 81.2 months (range 4.9–10.9 years)	Vocabulary age equivalent in months = 71.9 (SD = 23.1), (range 28–126) (PPVT-R)	Accuracy: word identification; word attack; (WRMT)	Reading-level	TD > DS: on nonword reading
DS	Roch et al. (2008) [49]	*n* = 12 DS *n* = 14 TD children	Mean age = 18 years 11 months (range 10.5–26.7) Mean age = 7 years 3 months (range 6.10–7.3)	Vocabulary: age equivalent in months = 97.17 (SD = 27.93) (BPVS-II)	Accuracy: word reading; nonword reading; Speed: word reading; nonword reading;	Reading-level	TD-DS = NS on speed nonword reading TD > DS: on accuracy nonword reading
DS	Nash et al. (2011) [50]	*n* = 13; 1 male, 12 females with DS *n* = 13 TD-reading ability, 2 males, 11 females *n* = 13 TD-comprehension ability, 6 males, 7 females *n* = 13 TD-poor comprehenders, 4 males, 9 females	Mean age (months) = 185.92 (range 11.4–19.3 years) Mean age (months) = 109.92 Mean age (months) = 103.23 Mean age (months) = 114.00	Vocabulary knowledge = 83.92 (SD = 14.63) (BPVS-II)	Accuracy: single word reading (BAS-II) nonword reading (GNRT)	Reading-level	TD > DS: on nonword reading
DS	Hulme et al. (2012) [51]	*n* = 49 DS, 22 males, 27 females *n* = 61 TD, 31 males, 30 girls	Mean age = 13.8 (range 8–17 years) Mean age = 6.1 (range 5–7 years)	Non-verbal ability; naming vocabulary; (BAS-II) (BPVS-II)	Accuracy: word reading (EWRT; BAS-II) nonword reading	Reading-level	TD > DS: on accuracy word reading TD > DS: on nonword reading
DS	Mengoni et al. (2014) [52]	*n* = 16; 5 males, 11 females with DS *n* = 16 TD males	Mean age = 13.8 (range 8–17 years) Mean age = 6.1 (range 5–7 years)	Matrices age-equivalent = 5 (SD = 1.03) (range 4–7.03) (WPPSI-III)	Accuracy: word reading (YARC) nonword reading (GNRT)	Reading-level	DS > TD = on word reading DS-TD = NS on nonword reading
DS	Lovell et al. (2016) [53]	*n* = 20 DS, 8 males, 12 females; *n* = 20 TD, 12 males, 8 females	Mean age = 16.16 (range 11–20 years) Mean age = 7.33 (range 5–9 years)	IQ = 47.75 (SD = 8.73) (KBIT-2)	Accuracy: word identification (WRMT-III) word attack (WRMT-III)	Reading-level	TD > DS: on nonword reading

**Abbreviations and Explanations**: TD: typically developing; SD: standard deviation; FSIQ: full scale intelligence quotient; WISC-R: Wechsler Intelligence Scale for Children-Revised; Brus-EMT: Brus-EénMinuut Test; WISC-III: Wechsler Intelligence Scale for Children-III; WIAT-II: Wechsler Individual Achievement Test-II; WRMT-R: Woodcock Reading Mastery Tests—Revised; Leiter-r: Leiter International Performance Scale-Revised; WJ-R: Woodcock-Johnson Tests of Academic Achievement–Revised; WORD: Wechsler Objective Reading Dimensions; GNRT: Graded Nonword Reading Test; DDE: Battery for Evaluating Developmental Dyslexia and Dysorthography; WISC: Wechsler Intelligence Scale for Children; PPVT: Peabody Picture Vocabulary Test; PROLEC-R: Baterìa de Evaluacìon de los Procesos Lectores-Revisada; BPVS: British Picture Vocabulary Scales; BAS: British Ability Scales; BAS-II: British Ability Scales-II; PPVT-R: Peabody Picture Vocabulary Test-Revised; BPVS-II: British Picture Vocabulary Scales-II; EWRT: Early Word Recognition Test; WPPSI-III: Wechsler Pre-school Primary Scale Intelligence-III; YARC: York Assessment Reading Comprehension; KBIT-2: Kaufman Brief Intelligence Test-2; WRMT-III: Woodcock Reading Mastery Tests-III.
